# Evaluation of CXCR1 as a possible diagnostic biomarker in acute appendicitis 

**Published:** 2020

**Authors:** Ensieh Khalkhal, Zahra Razzaghi, Alireza Akbarzadeh Baghban, Nosratollah Naderi, Mostafa Rezaei-Tavirani, Majid Rezaei-Tavirani

**Affiliations:** 1 *Proteomics Research Center, Faculty of Paramedical Sciences, Shahid Beheshti University of Medical Sciences, Tehran, Iran*; 2 *Laser Application in Medical Sciences Research Center, Shahid Beheshti University of Medical Sciences, Tehran, Iran*; 3 *Proteomics Research Center, School of Rehabilitation, Shahid Beheshti University of Medical Sciences, Tehran, Iran*; 4 *Gastroenterology and Liver Diseases Research Center, Research Institute for Gastroenterology and Liver Diseases, Shahid Beheshti University of Medical Sciences, Tehran, Iran*; 5 *Firoozabadi Hospital, Faculty of Medicine, Iran University of Medical Sciences, Tehran, Iran*

**Keywords:** Acute appendicitis, Biomarker, Diagnosis

## Abstract

**Aim:**

The present study was conducted to determine the genes with common expression in blood and appendix tissue samples in order to introduce them as possible diagnostic biomarkers.

**Background:**

Diagnosis of acute appendicitis (AA) without applying computed tomographytomography (CT), subjecting the patient to significant radiation, can be surprisingly difficult. Blood circulation may have conscious alterations in its RNA, protein, or metabolite composition.

**Methods:**

The genes related to appendix tissue and blood samples of the patients with AA were extracted from public databases. Fold change (FC) ≥ 2 in blood and FC ≥ 5 in appendix tissue samples were considered to screen differentially expressed genes (DEGs). A protein-protein interaction network was organized using the search tool for retrieval of interacting genes and proteins (STRING) database as a plugin of Cytoscape software version 3.6.0. The main genes were enriched by DAVID Bioinformatics Resources to find the related biochemical pathways.

**Results:**

Among the DEGs in blood and appendix tissue samples, C-X-C motif chemokine receptor 1(CXCR1), leukocyte immunoglobulin-like receptor A3 (LILRA3), low-affinity immunoglobulin gamma Fc region receptor III (FCGR3), and superoxide dismutase 2(SOD2) were common in both sources. CXCR1 was found as only hub gene upregulated in both blood and tissue of the patients with AA compared to controls and those with other abdominal pain.

**Conclusion:**

CXCR1, FCGR3, LILRA3, and SOD2 were determined as a suitable possible biomarker panel for diagnosis of AA disease.

## Introduction

 Acute appendicitis (AA) is one of the major causes of abdominal pain requiring urgent abdominal surgery. AA is characterized by mucosal ischemia caused by continuation of mucosal secretion in the form of distal luminal obstruction of appendix. So that, the amount of mucosal complex is increased inside the lumen leading to compression of the veins and because the lumen pressure exceeds 85 mm Hg, the veins are thrombosed. Also, venous congestion and obstruction wastes are increased (1,2). 

About 10% of people refer to the emergency department because of abdominal pain annually and incidence of AA is increasing (3, 4). AA symptoms are diffuse abdominal pain, nausea, and vomiting after several hours of topical pain. These are classically present in only one-third of the patients because of variety and extent of symptoms in AA and similarity in onset of symptoms to many abdominal diseases. AA is diagnosed based on complete physical examination and laboratory tests, the increased leukocyte and neutrophil counts, abdominal radiography ,and computed tomography(CT) scan (5). AA diagnosis is sometimes accompanied with difficulty and delay. Symptoms for patients with AA can also be seen in many abdominal diseases, such as gastritis, abdominal lymphadenitis, ovarian cyst complications in women, acute salpingitis, intestinal and parasitic infections, kidney stones, and urinary tract infections. Appendicitis surgery is the most common threatening emergency. Many of these diseases do not require surgery (6, 7). In the world, a small but significant proportion of surgeries are unnecessary. Due to AA misdiagnosis, 17-28% of appendix surgeries in the United States and Western Europe involving elimination of non-inflammatory lesions are mistakenly done so the patient undergoes postoperative complications (8-10). Despite high prevalence of AA, diagnosis of AA is still a challenge. Therefore, some paraclinical procedures can be helpful and rapid diagnosis of AA results in significant reduction in mortality and morbidity rates. In such circumstances, efforts to introduce simple, accurate, non-invasive and harmless diagnostic tools will be useful and effective. Therefore, this study was performed to determine a possible common diagnostic biomarker in blood and appendix tissue samples. 

## Methods

The keywords including “acute appendicitis”, “biomarkers”, and “diagnosis” were searched in the national center for biotechnology information (NCBI) and Google Scholar databases to find the proteomic and microarray-based papers about AA in the online journals published from 1990 until 2019. The microarray data were collected from public databases and gene expression databases. The differentially expressed genes (DEGs) involved in AA compared to healthy controls or patients with other abdominal pain obtained through literature survey, an experimental study, or database were combined.

All the collected DEGs of appendix tissue samples relative to those of the controls and also DEGs of blood samples compared to those of the controls were determined. Expression of AA-related genes in the appendix tissue samples was evaluated and then, the same set of genes was evaluated in blood. Fold change (FC)≥2 in blood and FC ≥5 in appendix tissue samples were considered to screen the studied DEGs. Protein-protein interaction (PPI) network for DEGs of tissue analysis was constructed using the search tool for retrieval of interacting genes and proteins (STRING) database as a plugin of Cytoscape software version 3.6.0 (11). Core component of the PPI network was analyzed by the Network Analyzer plug-in from Cytoscape software. The most important topological properties of PPI networks҆ nodes (degree value) were considered for ranking the network nodes. Over 20% of genes based on degree values were selected as hub genes. 

Common DEGs between tissue and blood samples were identified and were enriched by DAVID Bioinformatics Resources for analysis of biological processes, molecular function, cellular component, and biochemical pathway. 

## Results

Integrated data provided through literature survey including an experimental study and data from databases indicated that 121 genes in tissue of the patients with AA and 35 genes in blood samples were differentially expressed compared to the controls (6, 12-14). In appendix tissue sample, 57 and 64 genes were up and downregulated, respectively and in blood samples, 18 and 17 genes were up and downregulated, respectively compared to the controls (Table1). 

**Figure 1:**
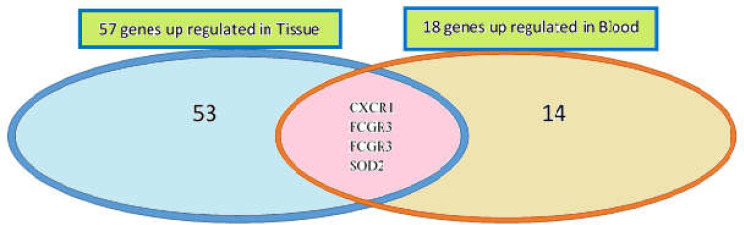
The number of common and differentially expressed genes in both blood and tissue samples of the patients with AA

Among the upregulated genes in tissue and blood samples; C-X-C motif chemokine receptor 1 (CXCR1), Fc fragment of IgG receptor III (FCGR3), leukocyte immunoglobulin-like receptor A3 (LILRA3), and superoxide dismutase 2 (SOD2) were common (Fig. 1). There were not common DEGs between the down-regulated DEGs of tissue and blood samples.

**Table 1:** List of the genes up or downregulated in appendix tissue and blood samples of the patients with AA

		**UP in tissue**	**Down in tissue**	**UP in blood**	**DOWN in blood**	**up in both source**
**1**		ANGPTL4	ADH1B	18S Rrna	DEFA1	CXCR1
**2**		APOBEC3A	ADH1C	28S rRNA	DEFA1B	LILRA3
**3**		AQP9	AKR1B10	ALPL	DEFA3	SOD2
**4**		BEST1	AQP8	C5orf32	LOC391370	FCGR3
**5**		CD163	ATP1A2	CA4	LOC644191	
**6**		CLEC5A	BCHE	CXCR1	NBPF10	
**7**		CLR1	CA2	CXCR2	RPL17L	
**8**		CSF2	CAPN6	CYSTM1	RPL21P28	
**9**		CSF3R	CCL15	FCGR3	RPL23	
**10**		CSPG2	CCL21	HLA-DRB5	RPL37A	
**11**		CXCL7	CHP2	LILRA3	RPLP1	
**12**		CXCL8	CLIC5	LOC100008588	RPS12P4	
**13**		CXCR1	CWH43	LOC100008589	RPS26	
**14**		FCGR2A	CXCL14	LOC100132394	RPS27P21	
**15**		FCGR3	DDC	LOC100134364	RPS27P29	
**16**		FPR1	DHRS11	NINJ1	RPS28	
**17**		G0S2	DPT	PROK2	RPS29P11	
**18**		GCSF	EPB41L4B	SOD2		
**19**		GPR43	ERAP1			
**20**		HK3	FCER2			
**21**		HPR	FRZB			
**22**		HSP70B	GIPC2			
**23**		HSPA6	GUCA2A			
**24**		IGSF5	HLF			
**25**		IL11	HMGCS2			
**26**		IL1RAP	HNF1B			
**27**		IL1RN	HPGD			
**28**		IL24	HSD11B2			
**29**		IL8	HSD17B2			
**30**		IL8RB	IGLC1			
**32**		INHBA	IGLJ3			
**33**		KCNJ15	KLF5			
**34**		LIL	LDB3			
**35**		LILRA3	LEFTY1			
**36**		LILRB1	LGALS4			
**37**		LILRB2	LRRC19			
**38**		LILRB3	LRRC31			
**39**		MARCO	MEP1A			
**40**		MGAM	MS4A12			
**41**		MMP1	MUC3B			
**42**		MMP10	NAT2			
**43**		N/A	NRIP2			
**44**		NCF2	NTRK2			
**45**		NFE2	NXPE4			
**46**		S100A12	PCK1			
**48**		S100A8	PLA2G2D			
**49**		S100A9	PTGDS			
**50**		SAA	SATB2			
**51**		SERPINE1	SELENBP1			
**52**		SOD2	SLC26A3			
**53**		SSA2	SLC4A4			
**54**		TFP12	SMPX			
**55**		TNFAIP6	SOSTDC1			
**56**		TNFRSF10	TMEM255A			
**57**		TRM1	TOX3			
**58**			UGT2A3			
**60**			UGT2B15			
**62**			UGT2B17			
**63**			USH1C			
**64**			VPREB3			

**Table 2:** Biological processes, cellular component, molecular function, and biochemical pathways related to the 4 common DEGs in both sources

BP	CXCR1
	Chemotaxis in dendritic cell , chemotaxis, inflammatory response, cell surface receptor signaling pathway, G-protein coupled receptor signaling pathway, receptor internalization, interleukin 8(IL-8) -mediated signaling pathway, chemokine-mediated signaling pathway,
CC	Plasma membrane, membrane, integral component of membrane
MF	IL-8 receptor activity, G-protein coupled receptor activity, chemokine receptor activity, IL-8 binding,
KEGG_PATHWAY	Cytokine-cytokine receptor interaction, chemokine signaling pathway, endocytosis, epithelial cell signaling in Helicobacter pylori infection
BP	FCGR3
	Immune response, Fc-gamma receptor signaling pathway involved in phagocytosis, regulation of immune response,
CC	Plasma membrane, external side of plasma membrane, integral component of membrane, extracellular exosome
MF	IgG binding
KEGG_PATHWAY	Phagosome, osteoclast differentiation, natural killer cell-mediated cytotoxicity, Leishmaniasis, Staphylococcus aureus infection, Tuberculosis, systemic lupus erythematosus
BP	LILRA3
	Adaptive immune response, defense response, signal transduction
CC	Extracellular region, plasma membrane
MF	Antigen binding, receptor activity
KEGG_PATHWAY	Osteoclast differentiation
BP	SOD2
	Regulation of blood pressure, response to reactive oxygen species, response to superoxide, oxygen homeostasis, removal of superoxide radicals, negative regulation of oxidative stress-induced intrinsic apoptotic signaling pathway, process, protein homotetramerization
CC	Mitochondria
MF	Oxidation-reduction activity, superoxide metabolic activity

**Figure 2:**
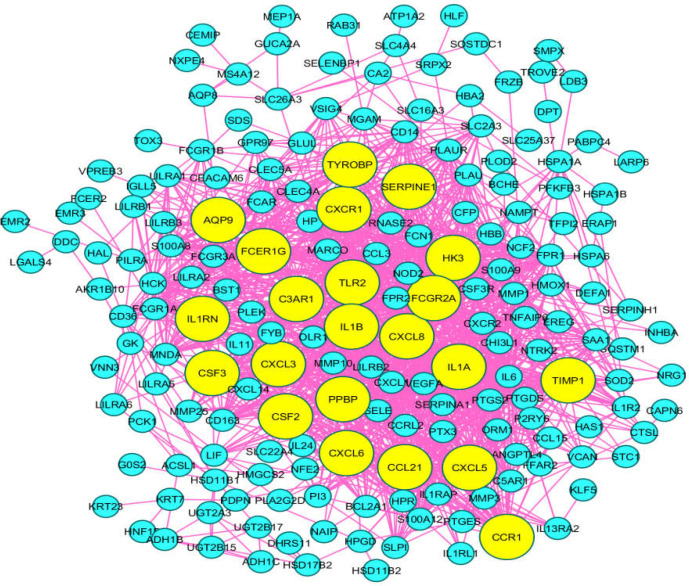
PPI network constructed by 121 DEGs extracted for AA tissue analysis. The hub nodes are presented in yellow color

Information regarding biological processes, cellular component, and molecular function related to the 4 common DEGs with similar expression change in both sources is shown in Table 2. As shown in Fig. 2, a total of 121 genes were included in the main connected component. The network was analyzed, and the nodes were laid out based on degree value. Top 20% of nodes based on the degree value including AQP9, C3AR1, CCL21, CCR1, CSF2, CSF3, CXCL3, CXCL5, CXCL6, CXCL8, CXCR1, FCER1G, FCGR2A, HK3, IL-1A, IL-1B, IL-1RN, PPBP, SERPINE1, TIMP1, TLR2, and TYROBP were selected as hub nodes. Among the hub genes, only CXCR1 had common expression with the four introduced and shared genes between tissue and blood samples

## Discussion

AA is the most common condition requiring urgent abdominal surgery (15). AA symptoms including diffuse abdominal pain, nausea, and vomiting after several hours of topical pain are present in only one-third of patients because of variety and extent of symptoms in AA and similarity in onset of symptoms to many abdominal diseases. AA is diagnosed based on complete physical examination and laboratory tests, abdominal radiography, and CT scan. It is difficult to diagnose AA without CT scan. In the cases where CT scan is not available, accurate diagnosis of AA can be challenging (10, 16). CT scan is now the "gold standard" for diagnosis of AA. For avoiding radiation in pregnant women, magnetic resonance imaging (MRI) and ultrasound sonography are an acceptable alternative to early diagnosis (5, 17). While CT scan is the most sensitive and specific diagnostic tool for diagnosis of AA and is used in approximately 98% of patients undergoing appendectomy in the United States, exposure to a carrier beam of CT scan is significant, and epidemiological data have suggested that radiation exposure can increase the risk of developing malignancy in the future (18-22). In such circumstances, it would be useful and effective to reduce the deleterious effects of CT scans by introducing simple, accurate, non-invasive, and harmless diagnostic tools.

Evaluating DEGs in the patients' appendix tissue and blood samples compared to controls and patients with other abdominal painshowed that CXCR1, FCGR3, LILRA3, and SOD2 were upregulated genes in the tissue and blood samples of the patients. Investigations have indicated that these DEGs are involved in inflammation, immunity, and infection (12).

CXCR1 (IL8 receptor α) is a chemokine receptore expressed in human leukocytes and infected epithelial cells (23, 24). It has high interaction with other proteins in PPI and is the only hub upregulated in both blood and appendix tissue samples. There are several documents about upregulation of IL-8 and its receptor (CXCR1) within the mucosa of the inflamed appendix and blood in the patients with AA compared to the patients without appendicitis (25-27). Therefore, high levels of IL-8 and CXCR1 are strongly associated with AA. 

FCGR3 is the important receptor for antibody-dependent natural killer cell-mediated cytotoxicity. Natural killer (NK) cells are innate lymphocytes providing defense against malignant or viral cells. In addition, NK cells mediate cellular antibody-dependent cytotoxicity. FCGR3 medites NK activity (28). In humans, there are two forms having 96% of sequence similarity in extracellular immunoglobulin binding regions. FCGR3A is expressed on mast cells, macrophages, and NK cells and is upregulated in appendix tissues of the patients with AA (6). FCGR3B is expressed only on neutrophils and is upregulated in blood of the patients with AA (29)(12).

 LILRA3 is a soluble receptor expressed in monocytes and B cells acting as modulator of immune reactions (30). It is a important regulator of immune cell activation by transforming opposing signals. It is widely present in the serum and appendix tissue of the patients with AA so it has strong clinical association with inflammatory diseases (31).

Results indicated that the 4 introduced DEGs were critical upregulated genes in the blood of the patients with AA therefore, they can be considered as a suitable diagnostic marker panel for AA. In this regard, the role of CXCR1 is prominent. In conclusion, 4 upregulated genes in blood of the patients with AA including CXCR1, FCGR3, LILRA3, and SOD2 are suggested as prominent DEGs, which are suitable to be considered as diagnostic biomarker candidates. However, the role and effect of CXCR1 was highlighted relative to the other 3 candidates
